# Antibacterial and Antimetastatic Potential of* Diospyros lycioides* Extract on Cervical Cancer Cells and Associated Pathogens

**DOI:** 10.1155/2016/5342082

**Published:** 2016-04-27

**Authors:** V. P. Bagla, V. Z. Lubisi, T. Ndiitwani, M. P. Mokgotho, L. Mampuru, V. Mbazima

**Affiliations:** Department of Biochemistry, Microbiology and Biotechnology, Faculty of Science and Agriculture, University of Limpopo, Turfloop Campus, Private Bag X1106, Sovenga, Limpopo 0727, South Africa

## Abstract

Cervical cancer is among the most prevalent forms of cancer in women worldwide.* Diospyros lycioides* was extracted using hexane, ethyl acetate, acetone, and methanol and finger print profiles were determined. The leaf material was tested for the presence of flavonoids, tannins, saponins, terpenoids, and cardiac glycosides using standard chemical methods and the presence of flavonoids and phenolics using thin layer chromatography. The total phenolic content was determined using Folin-Ciocalteu procedure. The four extracts were tested for antibacterial activity using bioautography against* Staphylococcus aureus*,* Enterococcus faecalis*,* Pseudomonas aeruginosa,* and* Escherichia coli*. The acetone extract with the highest number of antibacterial and antioxidant compounds was assessed for its cytotoxicity on BUD-8 cells using the real-time xCELLigence system and its potential effects on metastatic cervical cancer (HeLa) cell migration and invasion were assessed using wound healing migration and invasion assays. The leaf extract tested positive for flavonoids, tannins, and terpenoids while the four different extracts tested in the antimicrobial assay contained constituents active against one or more of the organisms tested, except* E*.* coli*. The cytotoxicity of the acetone extract in real-time was concentration-dependent with potent ability to suppress the migration and invasion of HeLa cells. The finding demonstrates the acetone extract to contain constituents with antibacterial and antimetastatic effects on cervical cancer cells.

## 1. Introduction

Medicinal plants act as one of nature's primary reservoirs since time immemorial as a source of potent pharmacological constituents for the treatment of various diseases. Multidrug resistance to antibacterial agents and the high incidences of various cancers on the other hand have grown at an alarming rate in recent years. Cervical cancer is the second leading form of cancer in South African women after breast cancer. It affects young women with resultant adverse effects such as infertility and impaired sexual quality [1].

The causative agent of cervical cancer is the Human Papilloma Virus (HPV) which is transmitted sexually, while* Staphylococcus aureus* and* Escherichia coli* are amongst the most common bacteria incriminated in vaginitis and associated cellular changes of the cervix [2]. Several other bacteria have also been implicated in chronic infections or are capable of producing toxins that influence the cell cycle resulting in altered cell growth [3], with a resultant DNA damage similar to that observed in apoptosis induced by carcinogens.

In South Africa, the bark and root decoctions of* Diospyros lycioides* are administered as treatment for bloody faeces and dysentery [4], while in Namibia the plant is used as a chewing stick [5]. Extract of this plant is also taken as a remedy for infertility in women [6], an associated problem in women with cervical cancer. Investigations of the antimicrobial activity of this plant have demonstrated the methanolic extract, from the twigs, to possess activity against common oral pathogens including* Streptococcus mutans* and* Porphyromonas gingivalis*, while isolated compounds have been found to possess marginal growth inhibitory activity against* Streptococcus sanguinis* and* S*.* mutans* [7].

*Diospyros lycioides* is known to contain some naphthoquinone constituents, including 7-methyljuglone, diospyrin, and isodisopyrin [8, 9]. Both 7-methyljuglone and diospyrin are reported to have a variety of pharmacological activities including anticancer activity [10, 11]. Evaluation of this plant in a recent study for its antiproliferative activity against MCF-7 breast cancer cells from our research group revealed the acetone leaf extract to induce apoptosis in a p53-dependent manner. The extract was also shown to upregulate Bax and downregulate Bcl-2 mRNA expression levels in MCF-7 breast cancer cells [12]. The current study was therefore aimed at investigating the antimetastatic effect of this plant on cervical cancer cells and the antibacterial activity against the pathogens that have been implicated in the condition. The extracts were also tested for the presence of selected phytochemical constituents.

## 2. Materials and Methods

### 2.1. Collection of Plant Material

The leaves of* D*.* lycioides* were collected from* Bolahlakgomo* area (*Lepelle*-*Nkumpi* Municipality, Limpopo Province, South Africa) and voucher specimen number UNIN 111076 is deposited at the Larry Leach Herbarium of the University of Limpopo. Leaves were separated from the stem and air-dried at room temperature in the dark. Dried plant material was ground into fine powder using a commercial blender.

### 2.2. Extraction of Plant Materials

The finely ground leaf material of* D*.* lycioides* (4 g) was serially extracted using 40 mL of each solvent, namely, hexane, acetone, ethyl acetate, and methanol for 3 h, 3 h, and 1 h at room temperature on a shaker (series 25 incubator shaker) set at 200 rpm. The extracts were filtered using Whatman filter paper number 3 into preweighed Erlenmeyer flasks, dried under a stream of air and masses obtained.

### 2.3. Phytochemical Screening

#### 2.3.1. Flavonoids

Dilute ammonia (5 mL) was added to a portion of an aqueous filtrate of the plant material. Concentrated sulphuric acid (1 mL) was added. A yellow colouration that disappears on standing indicates the presence of flavonoids [13].

#### 2.3.2. Tannins

About 0.5 g of the plant material was boiled in 10 mL of water in a test tube and filtered. A few drops of 0.1% of ferric chloride solution were added. A blue-black colouration indicated the presence of tannins [13].

#### 2.3.3. Saponins

To a 0.5 g of plant material, 5 mL of distilled water was added in a test tube. The solution was shaken vigorously and observed for a stable persistent froth [13].

#### 2.3.4. Terpenoids

To a 0.5 g of plant material, 2 mL of chloroform was added. Concentrated sulphuric acid (3 mL) was carefully added to form a layer. A reddish brown colouration at the interface indicated the presence of terpenoids [13].

#### 2.3.5. Cardiac Glycosides

To a 0.5 g of plant material dissolved in 5 mL of water, 2 mL of glacial acetic acid containing one drop of ferric chloride solution was added. About 1 mL of concentrated sulphuric acid was also added and a brown ring at the interface was observed for the presence of cardiac glycosides [13].

### 2.4. TLC Fingerprint Profile of* D*.* lycioides* Extracts

Thin layer chromatographic profile of the hexane, ethyl acetate, acetone, and methanol leaf extracts dissolved in acetone was analysed on an aluminium backed thin layer chromatography (TLC) plates (Fluka, silica gel F25). Stock solutions of 10 mg/mL each were made and 10 *μ*L of the extracts was spotted on TLC plates. TLC plates were developed in a saturated chamber using three mobile phases of different polarities, namely, benzene/ethanol/ammonia hydroxide [BEA, 5 : 4 : 1] (nonpolar/basic), chloroform/ethyl acetate/formic acid [CEF, 5 : 4 : 1] (intermediate polarity/acidic), and ethyl acetate/methanol/water [EMW, 4 : 1 : 5] (polar/neutral). TLC plates were sprayed with vanillin-sulphuric acid [0.1 g vanillin (Sigma®), 28 methanol, and 1 mL sulphuric acid] and heated at 110°C for optimal colour development [14]. For visualization of phenolic compounds, plates were sprayed with FeCl_3_ (2% in ethanol) and aromatics were viewed by spraying with iodine vapour.

### 2.5. Qualitative DPPH Assay on TLC Plates (Antioxidant Activity)

The TLC plates were prepared as described in methods for TLC finger print profile. All plates were dried in the fume hood before visualization. To detect compounds with antioxidant activity, the chromatographs were sprayed with 0.1% of 2,2-diphenyl-1-picrylhydrazyl (DPPH) (Sigma). The presence of yellow spots against purple background indicates the presence of antioxidant constituent [15].

### 2.6. Qualitative Detection of Phenolic Compounds Using TLC

TLC plates were prepared and eluted in the eluent systems as described methods for TLC finger print profile. Eluted plates were prayed with FeCl_3_. Grey or black bands indicate the presence of phenolic compounds.

### 2.7. Quantitative Detection of Flavonoids Using TLC

TLC plates were prepared and eluted in the eluent systems as described methods for TLC finger print profile. The flavonoid constituents were visualized using 10% antimony (III) chloride in chloroform. Yellow bands indicate the presence of flavonoids.

### 2.8. Detection of Starch and Glycogen Using TLC

TLC plates were prepared and eluted in the eluent systems as described methods for TLC finger print profile. Starch and glycogen constituents were visualized using iodine vapour indicated by the presence of blue bands.

### 2.9. Total Phenolic Content Assay

The total phenolic contents of* D*.* lycioides* extracts were determined by using the Folin-Ciocalteu assay. Hexane, ethyl acetate, acetone, and methanol extracts or standard solution of gallic acid (0.02, 0.04, 0.06, 0.08, and 0.10 mg/mL) was added to a 25 mL test tube, containing 9 mL of distilled water. The blank was prepared using water. One millilitre of Folin-Ciocalteu's phenol reagent was added to the mixture and shaken. After 5 min, 10 mL of 7% Na_2_CO_3_ solution was added to the mixture. The mixture was adjusted with water to a final volume of 25 mL. After incubation for 90 min at room temperature, the absorbance was read at 750 nm. Total phenolic content was expressed as mg/mL [16].

### 2.10. Bioautography

The TLC plates were prepared as described in Section 2.4 without spraying with vanillin-sulphuric acid and dried for 7 days under a stream of air to remove residual solvents. Plates were sprayed with 50 mL of concentrated suspension of fresh bacteria cultures, namely,* Staphylococcus aureus* (ATCC 29213),* Enterococcus faecalis* (ATCC 29212), and* Pseudomonas aeruginosa* (ATCC 27853), and incubated overnight at 37°C at 100% relative humidity. After incubation, plates were sprayed with 2 mg/mL solution of* p*-iodonitrotetrazolium chloride (INT) (Sigma). Retardation factor (*R*_*f*_) values of inhibitory zones, depicted as white areas [17], where reduction of INT to formazan did not occur, were recorded.

### 2.11. Cytotoxicity Assay Using xCELLigence System

BUD-8 cells were grown in Dulbecco's modified Eagle's medium (DMEM) (Gibco®) containing 10% fetal bovine serum (FBS) (Gibco) until about 75% confluent. Cells (100 *μ*L) at a density of 4000 cells/mL were seeded in each well of the E-plate 96 and incubated overnight. The cells were then exposed to various concentrations (5, 10, 100, 500, and 1000 *μ*g/mL) of* D*.* lycioides* acetone extract. Acetone extract was chosen for toxicity studies due to the presence of high number of antioxidant and antibacterial compounds active against tested pathogens. Controls received either 7.4 *μ*g/mL curcumin (positive control) or DMSO (negative control) at a final concentration of 0.05%. All experiments were run for 24 h.

### 2.12. Wound Healing Assay

The effect of the noncytotoxic concentration (150 and 300 *μ*g/mL) of the acetone extract of* D*.* lycioides* on mobility of HeLa cells was assayed using the wound healing assay. Cells suspended in DMEM containing 10% FBS were seeded in 12-well plates and incubated at 37°C for 24 h for cells to attain 90% confluence. A sterile plastic pipette tip was later used to create a linear wound across the centre of the cell monolayers. Cells were then washed twice with DMEM to remove detached cells and debris. The cell monolayers were then exposed to extracts at varying concentrations (0, 150, and 300 *μ*g/mL). Curcumin (7.4 *μ*g/mL) was used as positive control. Wound closure was monitored at 0, 6, and 24 h by photographing the monolayers under an inverted phase-contrast microscope (Nikon Eclipse, Ti series, USA) at 10x magnification.

### 2.13. Invasion Assay

Invasion assay was employed to determine the effect of the noncytotoxic concentrations of acetone extract of* D*.* lycioides* on HeLa cell invasion. Cells were treated with different concentrations of the extract (0, 150, and 300 *μ*g/mL) in serum-free DMEM. The cells were then seeded in cell culture inserts (8 *μ*m pore size) coated with 1 mg/mL of matrigel (BD Biosciences, USA). The inserts were then placed in 24-well plates filled with DMEM supplemented with 10% FBS (chemoattractant). The plates were incubated at 37°C for 24 and 48 h. Nonmigrating cells were wiped with a cotton-tipped swab, and invading cells were fixed with 80% methanol and stained with 0.5% crystal violet and photographed with a phase-contrast microscope at 10x magnification.

## 3. Results

### 3.1. TLC-Fingerprints of Extracts of* D. lycioides*

**Figure 1:**
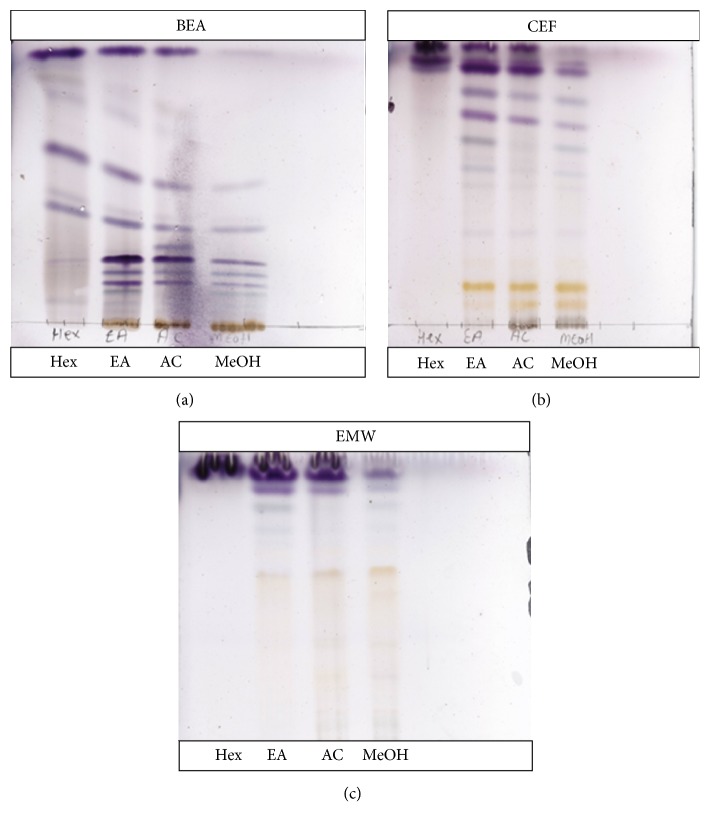
Thin layer chromatograms of hexane, ethyl acetate, acetone, and methanol extracts obtained from* D*.* lycioides* eluted in BEA (a), CEF (b), and EMW (c) mobile phases. The chromatograms were sprayed with vanillin/H_2_SO_4_ and heated at 110°C for colour development. Hex: hexane, EA: ethyl acetate, AC: acetone, and MeOH: methanol.

TLC profile of extracts from* D. lycioides* leaves showing different compounds that were separated using different mobile phases is presented in Figure 1. The TLC plates were developed using vanillin/H_2_SO_4_ and heated at 110°C. The movement of compound from the base of the TLC plates was dependent on the polarity of the solvent system used. More bands reacted with vanillin/H_2_SO_4_ with most of the separated constituents having a purple colour. With vanillin/H_2_SO_4_ spray, steroids, higher alcohols, phenols, and essential oils are likely to be detected which is suggestive of the presence of these groups of compounds in the extracts. Only few bands on reaction with the reagent did not produce coloured bands in all the mobile phases. Ethyl acetate (EA), acetone (AC), and methanol (MeOH) extracts showed the presence of almost similar compounds in all three mobile phases (Figures 1(a), 1(b), and 1(c)). Hexane extract showed a good separation of compounds only in the nonpolar (BEA) mobile phase (Figure 1(a)). Intermediate mobile phase (Figure 1(b)) showed more compounds compared to the other two systems, possibly because of the type of compounds that were well separated in the polar and acidic eluent system [18].

### 3.2. Test for the Presence of Antioxidant Constituents on TLC

**Figure 2:**
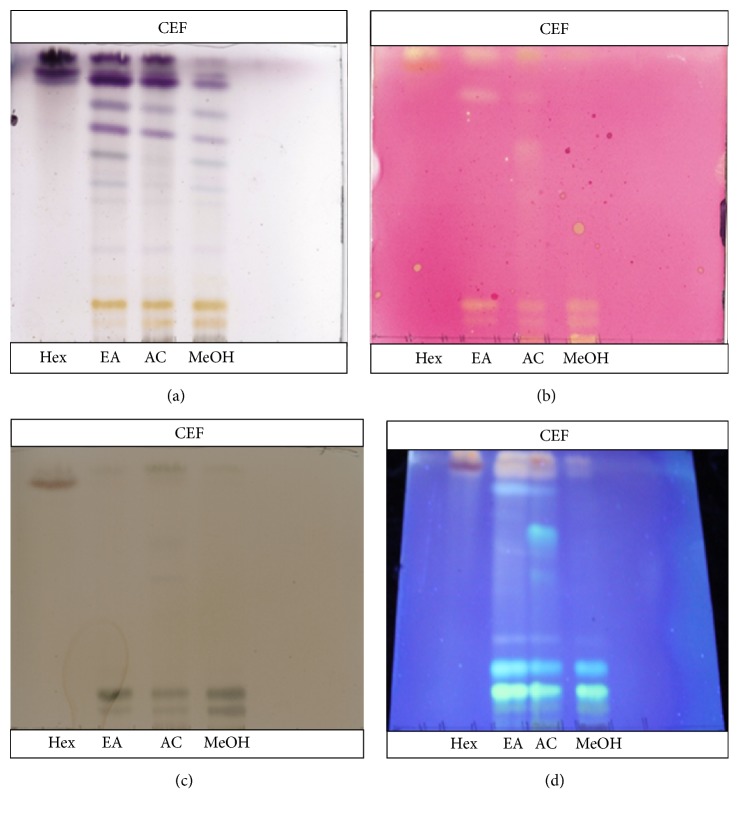
TLC finger print profiles (a), presence of antioxidant constituents (b), presence of phenolic compounds (c), and presence of flavonoids (d) eluted in CEF. Hex: hexane, EA: ethyl acetate, AC: acetone, and MeOH: methanol.

A representative TLC profile of different extracts of* D*.* lycioides* leaves eluted in CEF showing different constituents with antioxidant activity is presented in Figure 2(b). Chromatographs were sprayed with 0.1% DPPH solution. The yellow bands against a purple background indicate the presence of constituents with antioxidant activity. All extracts showed the presence of antioxidant constituents after spraying the chromatogram with DPPH. Hexane extract showed the least antioxidant active compounds while ethyl acetate and acetone showed more active compounds, especially in the intermediate mobile system. Most of the antioxidant compounds in ethyl acetate, acetone, and methanol could not move from the base of TLC plates when eluted in BEA solvent system, suggestive of the high polar nature of the constituent compounds.

The *R*_*f*_ values of constituents with antioxidant activity are presented in Table 1. With the hexane extract, antioxidant compounds migrated the most in the nonpolar and intermediate mobile phases, while acetone extract had more active compounds with *R*_*f*_ values ranging from 0 to 0.93 in both intermediate and polar mobile phases.

The phenolic constituents were visualized by spraying the eluted plates with FeCl_3_. Grey or black bands indicated the presence of phenolic compounds (Figure 2(c)). The compounds that tested positive for phenolics were consistent with those detected on qualitative analysis of antioxidant activity using DPPH on TLC, signifying that the antioxidant constituents in these extracts are generally phenolics. Flavonoid constituents on the other hand were visualized using 10% antimony (III) chloride in chloroform. Yellow bands indicated the presence of flavonoids (Figure 2(d)).

### 3.3. Evaluation of Total Phenolic Content in the Different Extracts

**Figure 3:**
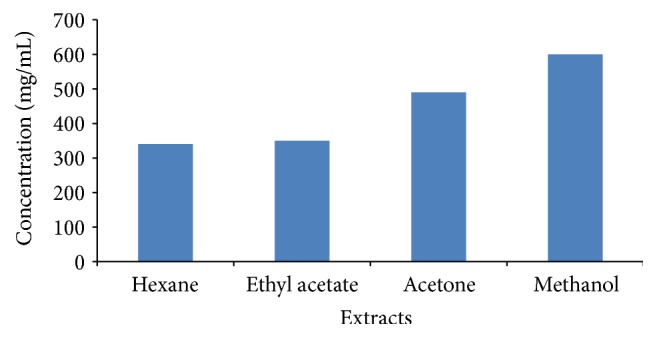
Phenolic content of* D*.* lycioides* leaves extracted from hexane, ethyl acetate, acetone, and methanol.

The phenolic content in the different extracts increased with increasing polarity of the extractant (Figure 3). Methanol extract showed the highest amount of phenolics followed by acetone. In hexane and ethyl acetate extracts, the difference was not significant. The amounts of phenolics did not correlate well with the presence of phenolics in the TLC-qualitative assay where acetone showed more bands compared to methanol.

### 3.4. Classes of Secondary Metabolite Contained in Extract

Test for the presence of phytochemical constituents in* D*.* lycioides* revealed the presence of tannins, terpenoids, flavonoids, and steroids and absence of saponins and cardiac glycosides. The presence of tannins and flavonoids has also been detected in this plant by other authors [19]. Flavonoids have been reported to have strong antimicrobial activity. Martini et al. [20] reported the antimicrobial activity of flavonoids against Gram-negative bacteria. Tannins are known to be toxic to fungi, bacteria, and yeast. One of the mechanisms by which tannins exhibit their antimicrobial activity is by inhibition of extracellular microbial enzymes [21]. Terpenoids are found in most plants and are known to be active against bacteria. Although their mechanism of action in inhibiting bacteria is not well understood, their action has been related to the disruption of bacterial cell membrane [22]. Taleb-Contini et al. [23] also reported steroids to have significant activity against* Staphylococcus aureus*,* Streptococcus faecalis,* and* Escherichia coli*.

### 3.5. Bioautography

**Figure 4:**
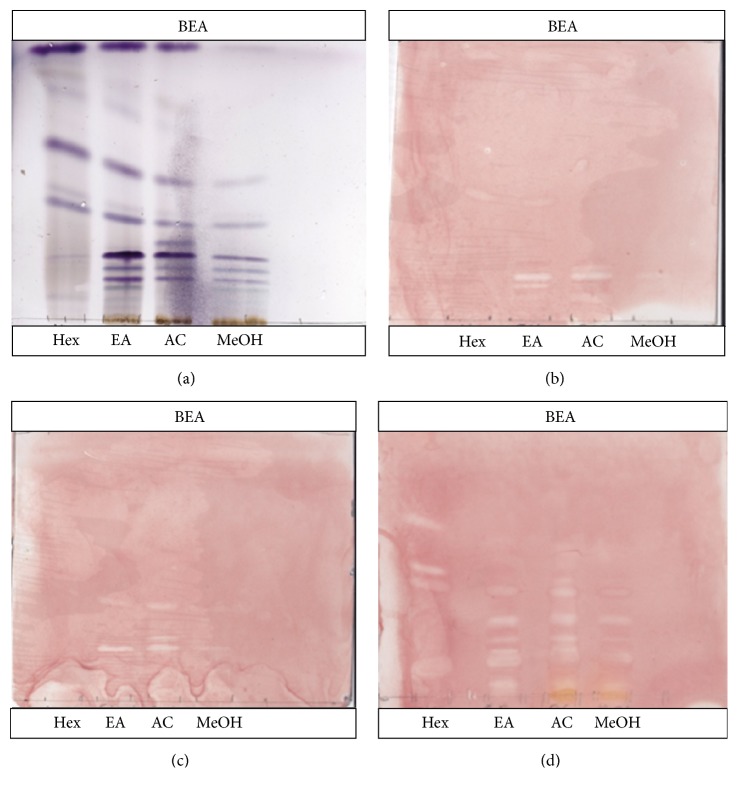
Representative bioautography of* D*.* lycioides* of hexane (hex), ethyl acetate (EA), acetone (AC), and methanol (MeOH) extracts eluted in BEA (a) and sprayed with* P*.* aeruginosa* (b),* S*.* aureus* (c), and* E*.* faecalis* (d).

Bioautography was used to screen for the presence of antibacterial compounds from* D*.* lycioides* extracts. Inhibition zones are indicated by the presence of white spot against a purple background. All extracts were unable to inhibit the growth of* E*.* coli* in all mobile phases (result not shown). The clear zone on TLC eluted in the representative bioautogram (Figure 4) indicates the presence of compounds within the different extracts with activity against the tested pathogen. Good growths of pathogens were observed with extracts that were eluted in BEA. The *R*_*f*_ value of compounds present in the different extracts against tested pathogens is presented in Table 2. More compounds were contained in the acetone and ethyl acetate extracts active against* S*.* aureus* and* E*.* faecalis*.

### 3.6. Monitoring of Cytotoxicity in Real-Time Using xCELLigence System

**Figure 5:**
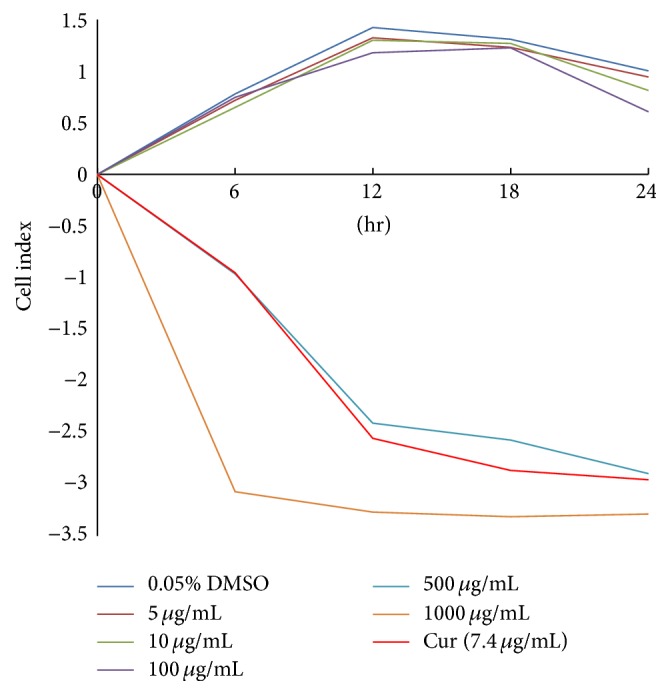
The cytotoxic effect of the acetone extract of* D*.* lycioides* on BUD-8 cells treated with increasing concentrations of crude acetone extract at 6 h interval as evaluated in real-time using xCELLigence system.

Acetone extract of* D*.* lycioides* that displayed the highest number of antibacterial and antioxidant compounds was found to be toxic to BUD-8 cell at concentrations of 500 and 1000 *μ*g/mL. Concentration ranges of 5 to 100 *μ*g/mL were not shown to be toxic at 18 h of exposure, with decrease in cell vailiblity (index) during proplonged exposure time beyond 18 h (Figure 5), with the cells exhibiting a similar trend (decrease in viability) beyond 18 h.

### 3.7. Effect of Acetone Extract on the Suppression of Migration of HeLa Cells

**Figure 6:**
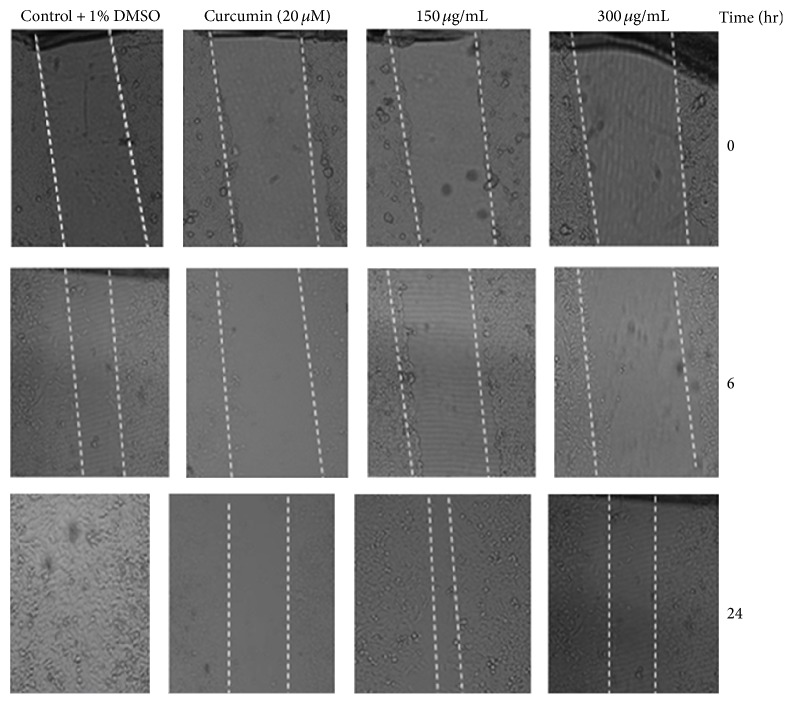
Inhibitory effect of the acetone extract of* D*.* lycioides* on the migration of HeLa cells. Confluent monolayers of cells were scarred, treated with 0, 150, and 300 *μ*g/mL of the extract and 7.4 *μ*g/mL of curcumin (positive control). Wound closure was monitored microscopically at 0, 6, and 24 h and photographed under a phase-contrast microscope at 10x magnification.

Since* S*.* aureus* and* E*.* coli* are amongst the most common bacteria implicated in vaginitis and associated cellular changes of the cervix; the acetone extract containing the highest number of antibacterial and antioxidant compounds was then tested at noncytotoxic concentrations (0, 150 and 300 *μ*g/mL) for its ability to inhibit the migration of HeLa cells. As shown in Figure 6, the extract was shown to suppress the migration of the cells in a concentration-dependent manner. Cells exposed to the acetone extract were seen to have a reduced ability to migrate and as such prevented the closure of wounds at the various concentrations and exposure times (Figure 6).

### 3.8. Suppression of the Invasive Ability of HeLa Cells

**Figure 7:**
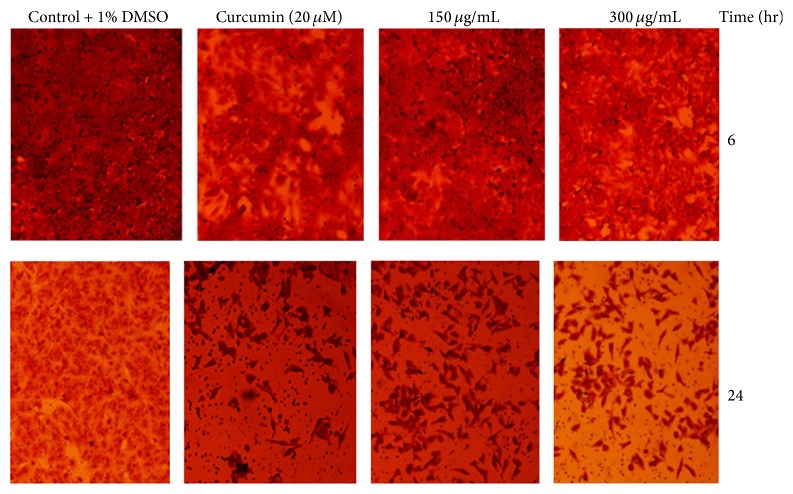
Anti-invasive potential of the acetone extract of* D*.* lycioides* on HeLa cell invasion. HeLa cells were treated with 0, 150, and 300 *μ*g/mL for 6 and 24 h. Curcumin (7.4 *μ*g/mL) was used as the positive control. Cells that penetrated through the matrigel membrane to the lower surface were stained with crystal violet and photographed with a phase-contrast microscope at 10x magnification.

The anti-invasive efficacy of* D. lycioides* acetone extract was assessed using the Boyden chamber coated with 1 mg/mL matrigel. Curcumin- and extract-treated cells were shown to considerably reduce the passage of cell through the matrigel as compared to untreated cells (Figure 7). Treatment with 300 *μ*g/mL of the extract resulted in greater reduction in invasive ability of the cells than 150 *μ*g/mL, implicative of the anti-invasive activity of the extract to be concentration-dependent with inhibitory activity at the highest concentration tested.

## 4. Discussion

*Diospyros lycioides* was selected for this study for screening of antibacterial activity because of its traditional use as a chewing stick against oral pathogens [5]. In this study, four solvents (hexane, ethyl acetate, acetone, and methanol) were used for extraction of plant material. Methanol, a polar solvent, was the best extracting solvent, signifying that most of the compounds contained in this plant are polar. TLC-fingerprints of* D*.* lycioides* extracts revealed the presence of various constituents. More compounds were observed in ethyl acetate extract, followed by acetone, while the intermediate polar solvent (CEF) was the best eluent system for the separation of constituents on TLC. Test for the presence of phytochemical constituents in* D*.* lycioides* revealed the presence of tannins, terpenoids, flavonoids, and steroids and negative for saponins and cardiac glycosides. Free radical scavenging activity of extracts on TLC indicates the presence of antioxidant constituents, most of which were of intermediate polarity. The ferric chloride reducing assay was used to detect the presence of phenolic compounds in the extracts. By comparing the bands that reacted with DPPH and those with ferric chloride and their *R*_*f*_ values, the study concludes with some degree of certainty that phenolic compounds are responsible for the observed antioxidant activities. The quality of antioxidant compounds was high in the methanol extract as compared to the other extracts. This group of compounds is considered to have extensive variety of physiological, antimicrobial, and antioxidant properties [24]. Flavonoids are known to exhibit their antimicrobial activity by penetrating the cell wall of the microorganism and have been reported to be active against Gram-negative bacteria [20].

The antibacterial activities of the extracts were evaluated using bioautography against* S*.* aureus*,* E*.* coli,* and* P*.* aeruginosa*. All the extracts were shown to contain one or more compounds active against tested pathogens except for* E*.* coli*. This finding was not surprising since previous studies [5] have reported the antimicrobial activity of the methanol extract of this plant against oral pathogens with subsequent isolation of four novel bioactive naphthalene glycosides, diospyroides-A,-B,-C, and -D. The lack of activity observed with this plant against* E*.* coli* is consistent with previous reports by Fawole et al. [19].

Indeed cancer is a multietiological disease and a manifold of processes maybe involved in cervical carcinogenesis. Tumors have a complex cellular ecology that establishes the malignant potential of the tumor. Since* Staphylococcus aureus* and* Escherichia coli* are amongst the most common bacteria that have been incriminated in vaginitis and associated cellular changes of the cervix [2], and given that macrophages also potentiate the propagation and establishment of metastatic cells and play a role in tumor initiation when inflammation is a causal factor [25, 26], we resolved to test the potential antimetastatic effect of the extract on cervical cancer cells (HeLa).

To archive this, the cytotoxic effects of the acetone extract which contains the highest number of antibacterial constituents and antioxidant activity were tested on BUD-8 cell (human fibroblast cells). Findings revealed the extract to decrease the cell index of normal cells (Bud-8 cells) with an increase in concentration (500 and 1000 *μ*g/mL) following analysis using the real-time-xCELLigence assay. An ideal antimetastatic agent ought to inhibit the metastatic events without affecting normal body cells. The relatively noncytotoxic concentrations of 150 *μ*g/mL and 300 *μ*g/mL of the extract were therefore chosen to access its inhibitory potential in subsequent metastatic-based assays on cervical cancer cells (HeLa cells), since preliminary studies of the extract on these cells in the MTT assay exhibited a >70% viability following 48 h of exposure (results not shown).

Exposure of cells to the acetone extract was shown to suppress the migration and invasive capability of HeLa cells in a time- and concentration-dependent manner. Tumor invasion requires degradation of basement membranes and thus remodelling of the extracellular matrix. Proteins that are mainly involved in degradation of the extracellular matrix are the matrix-metalloproteases (MMP). MMP-9 and MMP-2 are highly expressed in cancer patients. Observed effects suggest the extract to contain substances that can exert their antimetastatic effects by downregulating the activity of MMP-2 and MMP-9. Further study on the effect of the extract on the expression profiles and enzymatic-inhibitory activity of these proteins is necessary.

## 5. Conclusions

Cervical cancer is a burden in women. The acetone leaf extract of* D*.* lycioides* was shown to contain a variety of compounds, most of which were phenolics and flavonoids, with antibacterial activity against* S*.* aureus* and* P*.* aeruginosa.* Cytotoxicity of the acetone extract was shown to be nontoxic to normal cell at concentrations below 300 *μ*g/mL, with potent antimetastatic effect in the suppression of invasion and migration of HeLa cells, a process that is mediated by the downregulation of MMP-9 and MMP-2. The indication of the presence of compounds in the acetone extracts that can suppress HeLa cell migration and invasion makes it a potential source of drug candidates that can interfere with the metastatic process. Work is ongoing to elucidate the mechanism(s) by which the observed effect(s) is or are asserted.

## Figures and Tables

**Table 1:** *R*_*f*_ values and number bands with antioxidant activity.

Extracts	BEA		CEF		EMW	
	Number of bands	*R*_*f*_ values	Number of bands	*R*_*f*_ values	Number of bands	*R*_*f*_ values
Hexane	1	0.9	1	0.94	0	—
Ethyl acetate	1	0.96	4	0.06; 0.12; 0.79; 0.93	2	0.55; 0.90
Acetone	1	0.94	6	0.06; 0.12; 0.6; 0.8; 0.93	4	0.54; 0.60; 0.83; 0.89
Methanol	1	0.94	4	0; 0.06; 0.12; 0.93	2	0.38; 0.53

**Table 2:** *R*_*f*_ values of active compounds with antimicrobial activity eluted in BEA.

Extracts	*P. aeruginosa*		*S. aureus*		*E. faecalis*	
	Number of bands	*R*_*f*_ value	Number of bands	*R*_*f*_ value	Number of bands	*R*_*f*_ value
Ethyl acetate	2	0.10; 0.16	4	0.16; 0.22; 0.27; 0.36	4	0.05; 0.15; 0.17; 0.24; 0.37
Acetone	2	0.12; 0.17	3	0.2; 0.35; 0.45	5	0.05; 0.16; 0.17; 0.24; 0.58
Methanol	—	—	3	0.16; 0.2; 0.27	4	0.05; 0.15; 0.17; 0.24
Hexane	—	—	—	—	4	0.10; 0.39; 0.46; 0.61

## Abbreviations

TLC: Thin layer chromatography
BEA: Benzene : ethanol : ammonia hydroxide
CEF: Chloroform : ethyl acetate : formic acid
EMW: Ethyl acetate : methanol : water
DPPH: 2,2-Diphenyl-1-picrylhydrazyl
FeCl_3_: Ferric chloride
Na_2_CO_3_: Sodium bicarbonate
ATCC: American type culture collection
*R*_*f*_: Retardation factor
MMP: Matrix-metalloproteinases.
